# Research on Lightweight Citrus Flowering Rate Statistical Model Combined with Anchor Frame Clustering Optimization

**DOI:** 10.3390/s21237929

**Published:** 2021-11-27

**Authors:** Jianqiang Lu, Weize Lin, Pingfu Chen, Yubin Lan, Xiaoling Deng, Hongyu Niu, Jiawei Mo, Jiaxing Li, Shengfu Luo

**Affiliations:** 1 School College of Electronic Engineering and School College of Artificial Intelligence, South China Agricultural University, Guangzhou 510642, China; ljq@scau.edu.cn (J.L.); wei_ze_lin@163.com (W.L.); chenpingfu273@163.com (P.C.); ylan@scau.edu.cn (Y.L.); niuhongyu0207@163.com (H.N.); jiaweimo@stu.scau.edu.cn (J.M.); lee740464450@163.com (J.L.); luo8732402@163.com (S.L.); 2 National International Joint Research Center of Precision Agriculture Aviation Application Technology, Guangzhou 510642, China; 3 Lingnan Modern Agriculture Guangdong Laboratory, Guangzhou 510642, China

**Keywords:** citrus flowering rate, light weight, deep learning, edge computing, YOLOv4

## Abstract

At present, learning-based citrus blossom recognition models based on deep learning are highly complicated and have a large number of parameters. In order to estimate citrus flower quantities in natural orchards, this study proposes a lightweight citrus flower recognition model based on improved YOLOv4. In order to compress the backbone network, we utilize MobileNetv3 as a feature extractor, combined with deep separable convolution for further acceleration. The Cutout data enhancement method is also introduced to simulate citrus in nature for data enhancement. The test results show that the improved model has an mAP of 84.84%, 22% smaller than that of YOLOv4, and approximately two times faster. Compared with the Faster R-CNN, the improved citrus flower rate statistical model proposed in this study has the advantages of less memory usage and fast detection speed under the premise of ensuring a certain accuracy. Therefore, our solution can be used as a reference for the edge detection of citrus flowering.

## 1. Introduction

The determination of the flowering period of citrus trees is an important phase for citrus cultivation, as different agricultural activities are required in different growth periods. The number of flowers within the statistical scales is the main factor for determining the flowering period of citrus. At present, the determination of the citrus flowering period is mainly measured by human beings. It is inefficient and easily interrupted by subjective factors of observers. In recent years, with the improvement of convolutional neural networks and the computing power of computers, image recognition based on deep learning has been used in pedestrian detection [1], vehicle detection [2,3], fruit picking [4,5,6,7,8], pest detection [9], etc. We believe that similar approaches can be adopted for estimating the numbers of citrus flowers.

Citrus flower number estimation uses computer vision methods to identify, detect, and count individual citrus flowers in the image data. Among them, deep learning models can be divided into those established by target detection algorithms and instance segmentation algorithms [10]. Deng Ying et al. [11] proposed a citrus flower recognition and flower detection method based on instance segmentation. This method is based on the optimized Mask R-CNN [12] model to detect and count the number of citrus flowers in the image. Mask R-CNN is an instance segmentation algorithm, which requires a high level of calculation. In fact, there is no direct correlation between the shape of citrus flowers and the number of flowers. Therefore, the target detection method is a better choice for the task of spending statistics. Target detection can be further divided into one-stage and two-stage models. The two-stage model is represented by the Faster R-CNN [13,14,15] series; the one-stage model is represented by the YOLO [16,17,18] series. Faster R-CNN has a slow inference speed and is large in size, which is not suitable for mobile and embedded devices, while YOLOv4 [19] has a video stream detection speed of 96 frames per second (FPS), which can achieve the real-time detection effect, which is suitable for practical applications and outdoor productions.

Target detection is among the most popular research topics and has always been the focus of research in the industry [20]. In recent years, a variety of detection networks have emerged, and the YOLO series has been widely recognized for its excellent performance. However, in industrial applications, the existing detection network is difficult to directly apply in the industry due to the limited resources of hardware equipment and high requirements for speed and computation [21]. Therefore, when deploying large models in resource-constrained devices, model compression is a common approach. Existing model compression mainly includes model pruning [22,23], knowledge distillation [24], and quantification [25,26]. Song H et al. [27] proposed a deep compression method to streamline the network by deleting redundant connections, keeping the most informative connections, and quantitatively analyzing the weights to share the same weights. This method can be used to reduce the weight parameter while ensuring accuracy. However, the training time has not yet been reduced. Additionally, the compressed weights also need to be retrained to ensure accuracy.

Another effective approach to lightweight detection networks is to use a more lightweight backbone network. The lightweight feature extraction network, which features SqueezeNet [28], ShuffleNet [29,30], EfficientNet [31,32], GhostNet [33], and MobileNet [34,35,36], uses a lightweight trunk network to improve computing speed while maintaining accuracy. Qin et al. proposed ThunderNet [37], a mobile-oriented target detection network that uses a lightweight feature extraction network, SNet (an optimized ShuffleNetV2 [30]), and a proposed context enhancement module to integrate local and global features. To enhance the network feature expression ability, the detection network can be applied on the ARM platform to achieve the real-time detection effect. In 2020, Bochkovskiy A et al. proposed the YOLOv4 target detection network, which has the advantage of achieving a balance between speed and accuracy. However, its backbone network (CSPDarknet53) has a deep layer, which still requires a large number of floating-point operations and storage space in operation, and the resulting energy consumption has high requirements for the energy supply of edge computing devices. Therefore, it is necessary to modify its backbone network. The MobileNet series has been widely used for its excellent performance in many lightweight feature extraction networks. Compared with ordinary convolution, the deep separable convolution method proposed in MobileNet can greatly reduce the amount of computation while ensuring accuracy. Pan S et al. [38] proposed a lightweight steel surface defect detection method based on improved YOLOv3. The method modified the backbone network of YOLOv3 to MobileNetv2 to reduce the weight of the model. The improved model can detect speed on Raspberry Pi 3B. Compared with YOLOv3 [12,39] and YOLOv3-tiny, it was increased by 2 and 46 times, respectively. However, the detected mAP value only reached 60.24%. The current version of MobileNet [34,35,36] has been iteratively developed into the third-generation MobileNetv3, and its detection speed and accuracy have also been improved. Therefore, this study used MobileNetv3 as the feature extraction network, based on the YOLOv4 framework, to achieve accurate statistics of the number of citrus flowers, and compared the improved network with the original YOLOv4, YOLOv4-tiny, and Faster R-CNN [12], from the mAP, F1 value, and detection speed. The results were analyzed using evaluation indicators, such as the parameter amount.

Our contributions are as follows:

1. We modified the backbone network of YOLOv4 to MobileNetv3 and applied the deep separable convolution method to the neck network, which greatly reduced the number of parameters, and the feasibility of this method was also proved in the ablation experiment.
2. We used many methods to improve the detection accuracy, such as the k-means clustering algorithm [40] and Cutout [41] data enhancement. The experimental results prove that these methods have good performance.
3. The improved YOLOv4 performed well in terms of citrus flowering statistics, and the real-time detection speed reached 11.3FPS, which meets the needs of practical applications.

## 2. Materials and Methods

### 2.1. Data Collection and Preprocessing

**Figure 1:**
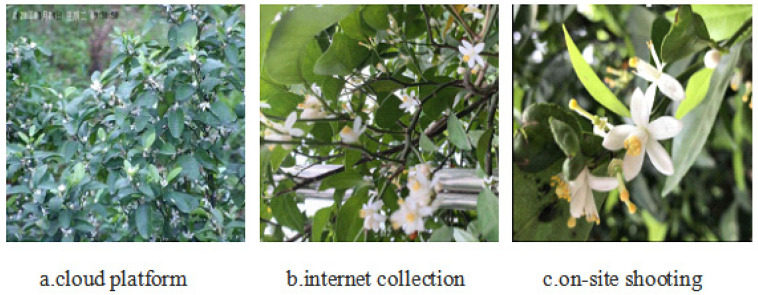
Images obtained in different ways.

The data were collected in different ways, such as on-site shooting, internet collection, and online shooting, on a smart agricultural cloud platform to obtain the required dataset; the samples from different sources are shown in Figure 1. The main image data were collected from Jinglongshui Village, Yangcun Town, Boluo County, Huizhou City, and Guangdong Province, China, using a fixed camera shooting and an internet collection on a smart agricultural cloud platform. Smartphones were also used to capture 2800 images from different shooting angles; crawler technology was adopted to download 170 citrus flower images from search engines, such as Baidu; after screening, 3000 pictures were obtained, including a total of 9237 real citrus flower sample sets. The LabelImg labeling tool was used to label all the data, where the flower was labeled as the “flower”, and the flower bud was labeled as the “bud”. As the data collection time was limited to 3–4 months, the sample size was not large enough, and the proportion of flowers in the data set was greater than the proportion of flower buds.

### 2.2. Data Enhancement

**Figure 2:**
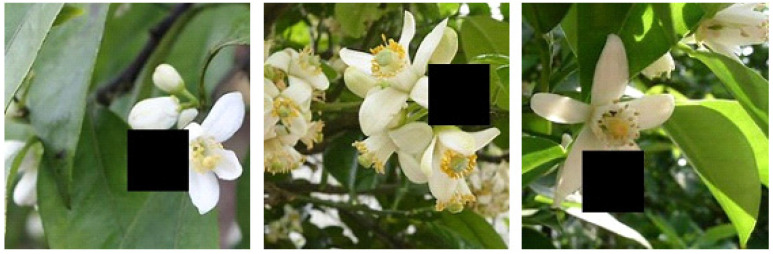
Figures after using the Cutout method.

To enlarge and balance the data set, the Skimage image processing library of Python was introduced to perform random angle flipping, cropping, mirroring, translation, brightness modification, and noise addition for the original 3000 pictures. Furthermore, a Cutout data enhancement method was adopted, which defined a rectangle with a size of 100 × 100 to occlude the input image and simulate a phenomenon in which flowers in natural orchards were blocked, as shown in Figure 2. Within the rectangle, all pixel values were filled with 0. Through the Cutout operation, the convolutional neural network can better obtain the global information of the image, instead of relying on a small number of specific visual features. Additionally, appropriately adjusting the proportion of categories, that is, increasing the expansion proportion of the pictures containing flower buds, was performed to balance the sample. Finally, the original data set was expanded to 9610, and the data set was divided into a training set, validating set and test set in the ratio of 7:2:1, respectively.

### 2.3. K-Means Border Clustering

**Figure 3:**
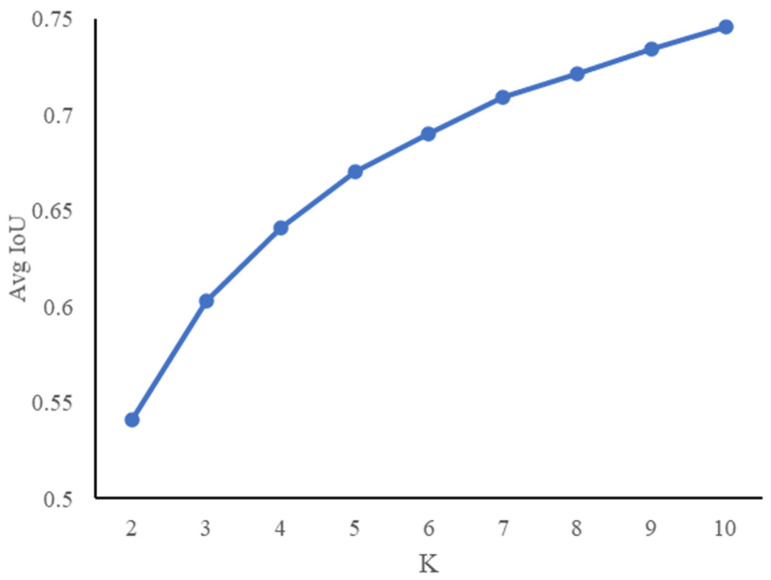
K-means clustering analysis result.

In YOLOv4, the concept of the anchor box is introduced, and the size of the anchor box is usually set to multiple fixed values. The selection of the size of the anchor box plays an important role in later object recognition. To improve the efficiency of model training, this study proposed the K-means clustering method to obtain the anchor box size suitable for the citrus flower target before training. Figure 3 shows the relationship between k and Avg IoU with the k range from 2 to 10. With the increase in the k value, the trend of Avg IoU was close to stable, and the point with the largest slope was considered as the best k value. Although an increase in the value of k also means an increase in the recall rate, the slope of the point will gradually decrease. In order to balance the recall rate and the best k value, k = 5 was selected as the number of anchor boxes. When the input image size was 608 × 608 pixels, the dimensions of these 5 anchor boxes were 23 × 22, 42 × 39, 64 × 62, 104 × 84, and 165 × 141, respectively.

### 2.4. Lightweight YOLOv4 Model

**Figure 4:**
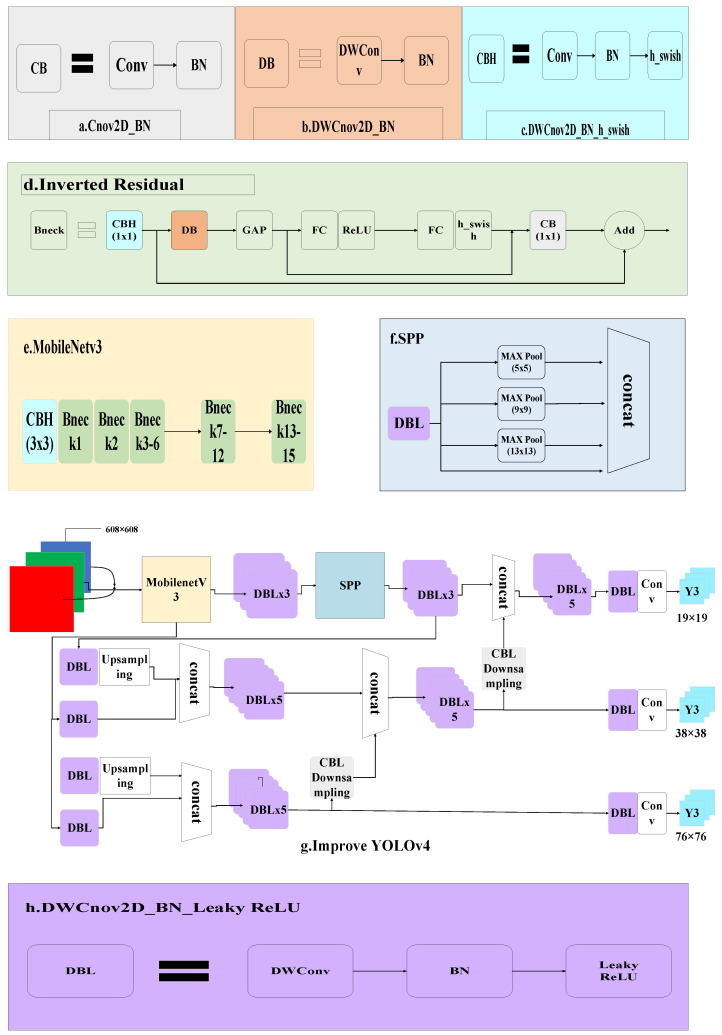
The network structure of improved YOLOv4.

The structure of YOLOv4 is composed of the backbone network CSPDarknet53, spatial pyramid pooling (SPP), path aggregation network (PANet), and YoLohead, with many network layers and a large number of model parameters. In order to speed up the detection of the network while maintaining the detection accuracy for edge computing, the original YOLOv4 was modified by replacing the backbone network with MobileNetv3 and replacing ordinary convolution with deep separable convolution in the neck network. The lightweight YOLOv4 network is shown in Figure 4.

In backbone network optimization, unlike the original backbone network CSPDarknet53, this study adopted MobileNetv3, which is composed of a series of neck structure blocks and inverted residual units instead of the residual component used by CSPDarknet53. The inverse residual component first uses pointwise convolution (PWC) to increase the number of dimensionality channels, then uses depthwise convolution (DWC) for feature extraction, and finally uses PWC for dimensionality reduction compression. This inverted bottleneck structure enables feature extraction to be performed in high dimensions, so as to extract more feature information and reduce the number of parameters while maintaining high precision.

In Figure 4, DWConv refers to the use of deep separable convolution; Mult refers to the depth multiplication of the one-dimensional matrix obtained through two full connection layers and the original matrix, that is, the application of an attention mechanism to each channel of the characteristic matrix; Concat-operation refers to the Concat operation in depth. The DBL (marked in purple) consists of a deep separable convolutional layer, batch normalization, and Leaky_ReLU activation functions. The CBH (marked in white) consists of a convolutional layer, batch normalization, and h_swish activation functions. The CB (marked in brown) consists of a convolutional layer and batch normalization. DB consists of a convolution layer and batch normalization. The CBL (marked in dark grey) module is composed of a convolutional layer (Conv), batch normalization (BN), and a leaky ReLU activation function.

**Figure 5:**
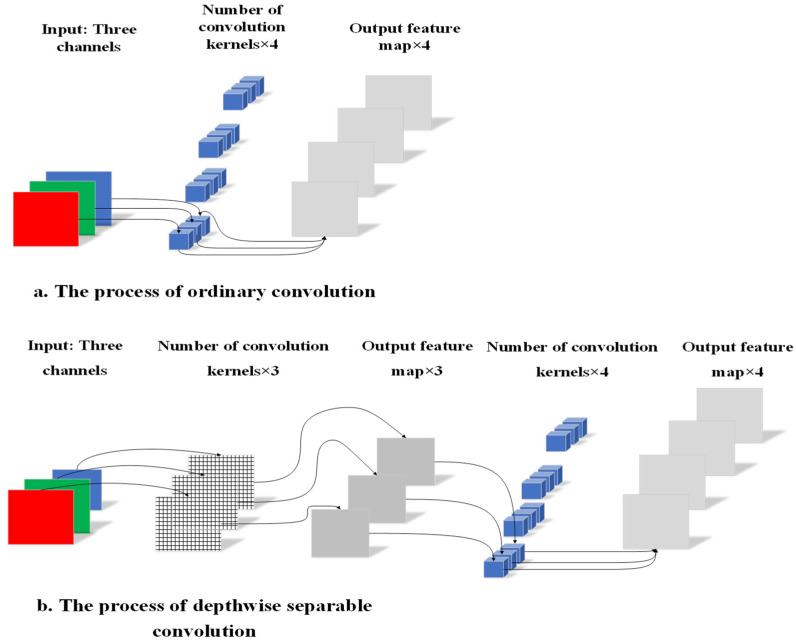
Ordinary convolution and depthwise separable convolution.

In the convolutional optimization of the neck network, deep separable convolution was applied to the neck network, which is composed of DWC and PWC. The channel number of the convolution kernel was equal to that of the input feature matrix, and the channel number of the output feature matrix was equal to one of the convolution kernels used. The number of channels of the DWC convolution kernel was one; that is, for the input feature layer, each channel used only one convolution kernel for deconvolution, so the number of output channels was also equal to the number of input channels. PWC used a 1 × 1 ordinary convolution kernel for convolution. After PWC, the number of channels of the output feature matrix was equal to the number of PWC convolution kernels used. Figure 5a,b show ordinary convolution and the process of DWC + PWC. Equations (1) and (2) are the parameter quantities of ordinary convolution and DW + PW, respectively, where *H_i_*, *W_i_*, and *D_i_* refer to the height, width, and depth of the input feature matrix, respectively; *H_k_* and *W_k_* refer to the height and width of the convolution kernel, respectively; *D_o_* refers to the depth of the output feature matrix.

(1) $$q_{1}=H_{i}\times W_{i}\times D_{i}\times H_{k}\times W_{k}+D_{i}\times D_{o}\times H_{k}\times W_{k}$$

(2) $$q_{2}=H_{i}\times W_{i}\times D_{i}\times D_{o}\times H_{k}\times W_{k}$$

From Equations (1) and (2), it can be concluded that the ratio of the calculation amount of the depth separable convolution and the standard convolution is as follows:

(3) $$\frac{H_{i}\times W_{i}\times D_{i}\times H_{k}\times W_{k}+D_{i}\times D_{o}\times H_{k}\times W_{k}}{H_{i}\times W_{i}\times D_{i}\times D_{o}\times H_{k}\times W_{k}}=\frac{1}{D_{o}}+\frac{1}{H_{i}\times W_{i}}$$

We studied the effect of the above methods on the number of parameters. When only the backbone network of YOLOv4 was replaced by MobileNetv3, the number of parameters was reduced by 93 MB, which is 38% less than the original. When the 3 × 3 ordinary convolution in the original YOLOv4 was modified into deeply separable convolution, the number of parameters was reduced by 108 MB, which is 44% less than the original. When replacing the backbone network of YOLOv4 with MobileNetv3 and using deep separable convolution in the neck, the number of parameters was reduced by 190 MB, which is 77% less than the original. It can be seen that modifying the backbone and using depth separable convolution in the neck can greatly reduce the parameters.

## 3. Experiments and Results

### 3.1. Experimental Environment Setting

**Figure 6:**
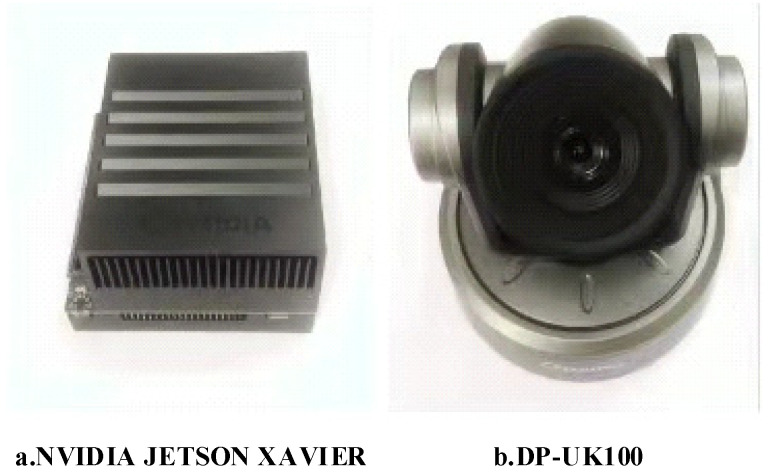
Test platform.

The training environment was composed of a processor (Intel Xeon CPU E5-2620 v4; Intel, Santa Clara, CA, USA) and Graphics Processing Unit (Nvidia Titan X; Nvidia, Campus in Arizona, USA). The reasoning environment adopted edge computing equipment [42,43] (Nvidia Jetson AGX Xavier; Nvidia, Campus in Arizona, USA) and a camera (DaiPu DP-UK100; DaiPu, Beijing, China), as shown in Figure 6. The software environment was composed of the Ubuntu18.04 operating system, Python programming language, Pycharm compilation environment, and Pytorch1.7. deep learning framework.

For comparison, Faster R-CNN [44], YOLOv4, and YOLOv4-tiny were adopted to compare their schemes. To speed up the convergence of the model, the transfer learning method [45,46] was adopted for the four models, and the weight files obtained from training on the PascalVOC data set were used for pre-training. Mosaic data enhancement was used in the training process; that is, four pictures were loaded at a time, and operations such as random rotation, color gamut change, and zooming were performed to regenerate new images. The regenerated images fused the background information of different images, reducing the dependence of the model on the location of the target and improving the generalization ability of the model.

The four networks on the same training set were trained in the same way. The input image size was 608 × 608, and each batch contained 32 images. The iteration time was set to 75,000. The initial learning rate was set to 0.001. The cosine annealing was used to adjust the learning rate, which made the model more diversified and produced better training effects.

### 3.2. Model Evaluation

The prediction accuracy rate (*P*), recall rate (*R*), average precision (mAP), and F1 value were used as evaluation indicators. The formulas of *P*, *R* mAP, and F_1_ scores are shown in Equations (4)–(8).

(4) $$P=\frac{TP}{TP+FP}$$

(5) $$R=\frac{TP}{TP+FN}$$

where *TP* refers to the number of positive samples that were predicted correctly, *FN* refers to the number of samples that were missed, *FP* refers to the number of samples that were wrongly detected, and *TN* refers to the number of negative samples that were predicted to be correct.

(6) AP=∫PRdr

(7) $$\mathrm{mAP}=\frac{1}{C}\sum_{i=1}^{C}AP_{i}$$

(8) $$F_{1}=2\times \frac{P\times R}{P+R}=\frac{2TP}{2TP+FP+FN}$$

where *C* refers to the number of categories, and *AP_i_* is the *AP* value of the *i*-th category.

### 3.3. Ablation Experiment

In order to verify the optimization effect of each module used in the improved YOLOvV4 on YOLOv4, this study conducted ablation experiments. First, the 3 × 3 convolution in the feature fusion part of YOLOv4 was replaced with a depth separable convolution; then, MobileNetv3 and tiny structures were added separately on the basis of the model; finally, the improved YOLOv4 algorithm was compared and verified. Table 1 shows the results of ablation experiments on the improved YOLOv4. In the table, MobileNetv3 and tiny represent the replacement of the CSPDarknet53 backbone network in YOLOv4 with the MobileNetv3 structure and the tiny structure, and dw represents the replacement of all 3 × 3 convolutions in the feature fusion network with depth separate convolution. The ablation experiments compared the performance with the mAP, parameter quantity, model size, and real-time monitoring FPS under various model structure combinations.

Compared with the 244 M size of the original YOLO V4, the model size of YOLO V4+dw decreased by 108 M after replacing the 3 × 3 convolution in the feature fusion network with the depth separable convolution, while the mAP only decreased by 1.6%, which proves the effectiveness of the separable convolution structure. After replacing CSPDarknet53 with MobileNetv3 and tiny separately in the model of YOLO V4+dw, the model size further decreased to only 53.7 and 22.4 M, respectively, but the mAP of the tiny scheme decreased to 64.42%, which does not meet the accuracy requirement of citrus flower detection; however, the mAP of the MobileNetv3 scheme was 84.84%, which only decreased by 2.56% and reached the requirement of citrus flower detection accuracy, which means that a high mAP can be guaranteed while greatly reducing the size of the model and the number of parameters.

**Figure 7:**
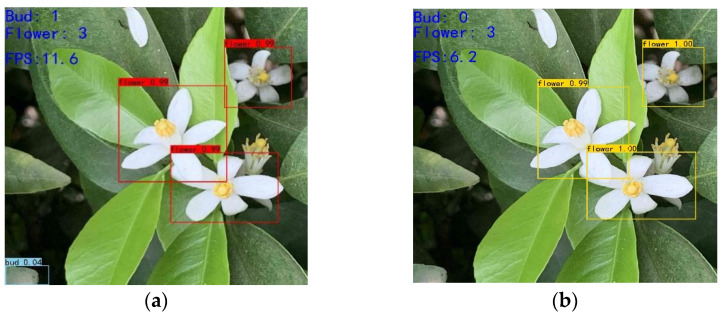
Comparison of real-time FPS detection between improved YOLOv4 and YOLOv4. (**a**) Improve YOLOv4 real-time FPS. (**b**) YOLOv4 real-time FPS.

The real-time detection speed comparison between the improved YOLOv4 and YOLOv4 is shown in Figure 7. The improved YOLOv4 real-time detection FPS was 11.6, while the real-time detection FPS of YOLOv4 was only 6.2; that is, the frame rate per second of the improved YOLO V4 was 46.5% higher than that of YOLOv4, greatly improving the real-time target detection speed.

### 3.4. Analysis of Training Loss and mAP

**Figure 8:**
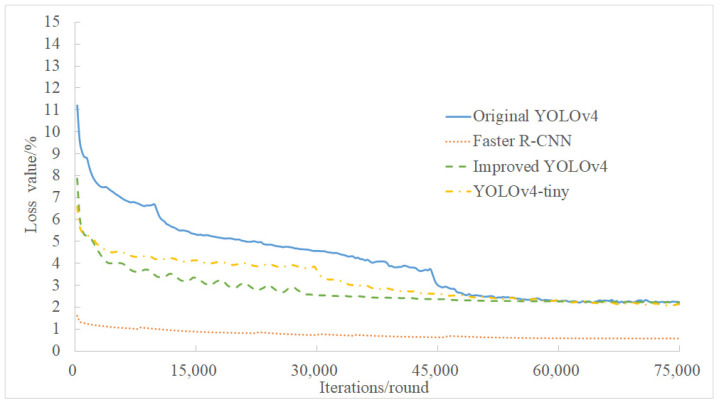
Loss value of the four models.

The overall loss values of the four models in Figure 8 show a downward trend, indicating that the fitting degree of the model to the characteristics of the citrus flower target gradually improved. The citrus flower detection model obtained by Faster R-CNN training had the smallest loss value and the highest degree of fitting to the training set. The improved YOLOv4 proposed in this study showed a more obvious decline in loss value and a faster convergence rate than the original YOLOv4, indicating that the improved YOLOv4 feature fitting is faster.

**Figure 9:**
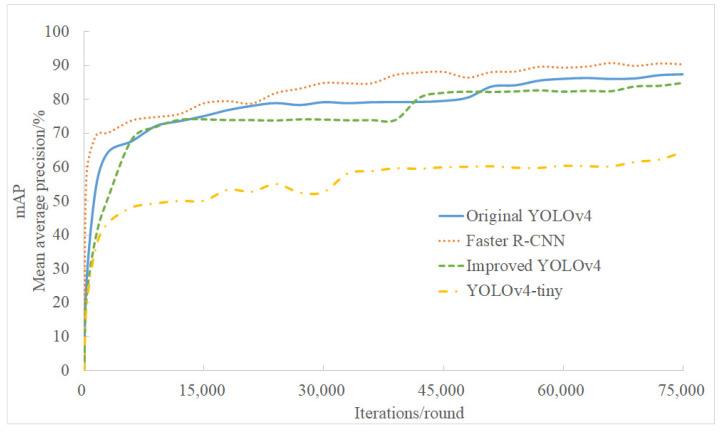
The mAP of the four models.

The changes in mAP of the four models with the increase in the number of iterations are shown in Figure 9. In the training, the mAP of the four models all showed an upward trend. Among them, Faster R-CNN performed best, and the improved YOLOv4 was similar to YOLOv4 and was significantly better than YOLOv4-tiny.

### 3.5. Comparison of Four Models

During the testing process, the weights of networks were saved every 3000 iterations, and the saved weights were tested and evaluated in turn. Table 2 shows the test results using the last saved weight. Compared with YOLOv4 and Faster R-CNN, the improved YOLOv4 reduced the mAP by only 2.56% and 5.43%, but its detection speed increased by 87% and 403%, respectively. The original YOLOv4 obtained a detection frame rate of only 6.4FPS, which could not achieve real-time detection when deploying edge computing devices. The speed of the improved YOLOv4 increased by 87.09%, basically realizing real-time detection, and the parameters were only 20% of YOLOv4. The parameters and detection speed of the improved YOLOv4 were slightly lower than those of the YOLOv4-tiny algorithm, but its mAP was 20.42% higher than that of the YOLOv4-tiny algorithm, and the false detection rate was high, which makes it difficult to meet the actual requirements. The improved YOLOv4 achieved a better balance between detection accuracy and light weight and achieved accurate detection while simplifying the model and improving the speed.

### 3.6. Testing Results under Different Citrus Flower Densities

In the actual orchard environment, the flower density and flower occlusion affect the recognition accuracy of the model. To test the improved YOLOv4’s detection performance under different citrus flower densities, this study used 150 sparse, medium, and dense citrus flower data sets for testing. The test results are shown in Table 3. In the case of sparse and medium citrus flowers, the mAP of the improved YOLOv4 reached 95.5% and 88.1%, and the F1 value reached 94% and 87.5%, indicating that the improved YOLOv4 has a strong detection performance. In the case of dense citrus flowers, their mAP and F1 values were relatively lower. This is due to the fact that MoileNetv3 has limited feature extraction capabilities, especially for small targets such as flower buds. One of the reasons for the missed inspection of flower buds is that the total number of flower buds was small, which led to insufficient features being learned by the model; another reason is that the very small targets were not difficult to label during manual labeling.

### 3.7. Testing Results under Different Environments

**Figure 10:**
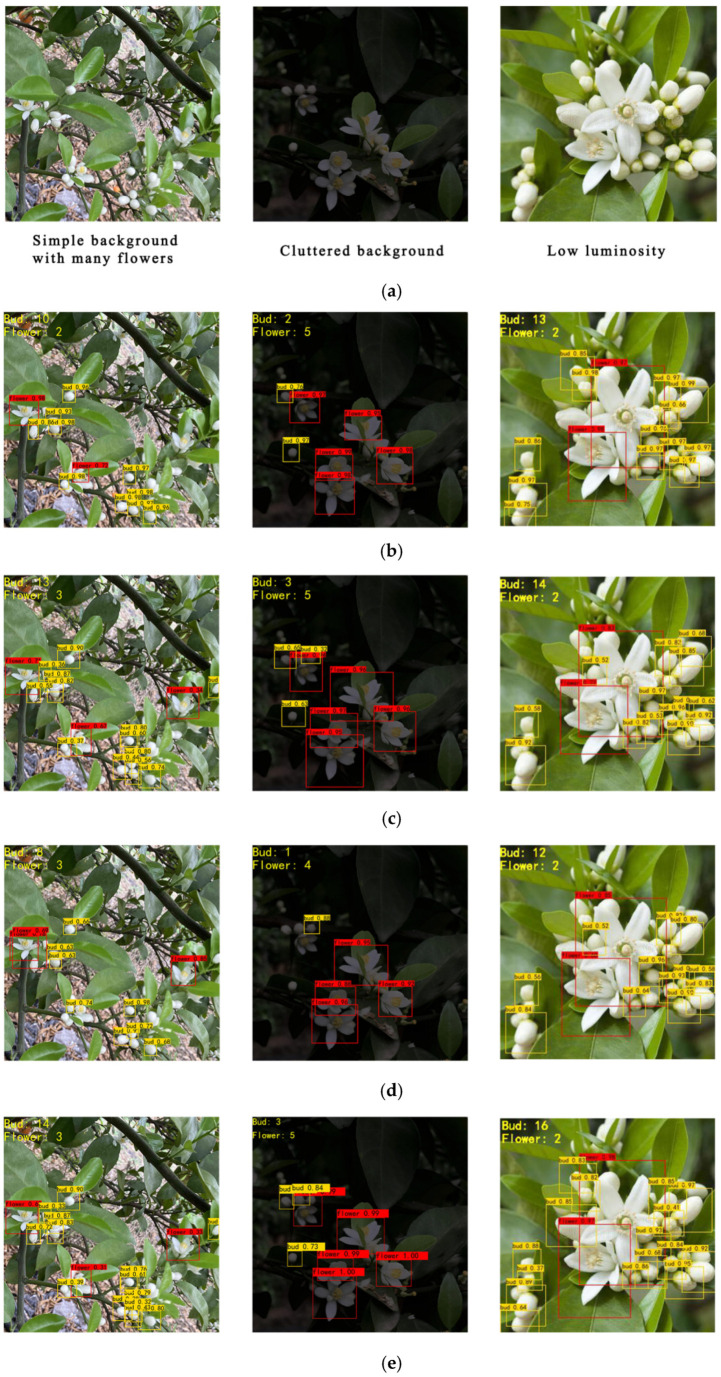
The detection effects of different models under different conditions. (**a**) Original images. (**b**) The detection results of YOLOv4. (**c**) The detection results of improved YOLOv4. (**d**) The detection re-sults of YOLOv4-tiny. (**e**) The detection results of Faster R-CNN.

In the natural environment, due to the influence of the climate and camera angle, the captured citrus flower images may be overexposed or not bright enough. To verify the detection effect of the improved YOLOv4 under special circumstances, this experiment selected three sets of images from the test set. The three sets of image data represent the simple background, cluttered background, and low luminosity, respectively. Figure 10 shows the effect of four models under the above conditions. The detection boxes labeled “flower” in the image indicate that the target detected is a citrus flower, and the detection boxes labeled “bud” indicate that the target detected is a citrus bud. The number of buds and flowers present in each image is shown in the upper left corner of each image. Compared with YOLOv4, the proposed improved YOLOv4 method in this study detected more buds for the simple background with many flowers and obtained a proximity effect for the low-luminosity image; compared with YOLOv4-tiny, the proposed method showed better detect performance, especially for low-luminosity cases; compared with Faster R-CNN, the proposed network is light and has a high speed, as shown in Table 1, but at the cost of a slightly inferior detection effect. From the comparison of Figure 9 and Table 1, a good compromise was achieved through the proposed method in this study.

## 4. Discussion and Conclusions

### 4.1. Discussion

Figure 7 shows that the improved YOLOv4 proposed in this study has faster convergence and feature-fitting speed than the original YOLOv4. The improved YOLOv4 has an accuracy rate of 84.84% and a detection speed of 11.6FPS, which meets the performance requirements of practical applications. As regards YOLO-tiny, the detection speed of YOLO-tiny is 23.5 FPS, which is faster than ours, but its accuracy is only 64.42% (20.42% lower). Our solution achieves a better balance between accuracy and speed. Furthermore, in Table 2, the tests under different densities (few, middle and intensive) show that the model accuracy is 87.4%, 84.84%, and 64.42%. Our model is good enough to be applied in automated agricultural robotics, but there is still room for improvement under highly intensive scenarios.

The K-means clustering unsupervised learning method used to obtain the anchor box size suitable for the citrus flower target improved the efficiency of model training. To classify data that are not known in advance into several categories, unsupervised learning can better perform data classification tasks. The data were aggregated into several groups through cluster analysis, and clustering does not require data training or learning. As shown in Figure 1, as the value of k increases, the value of IOU also increases, but the larger the k-value, the greater the complexity of the model. In order to balance the accuracy and complexity of the model, k = 5 was selected (the point with the largest slope) to be used as the number of anchor boxes. In this study, there are still shortcomings in the application of clustering unsupervised learning methods. In deep learning model training, the volume of data required for training is massive, and some invalid data will interfere with the accuracy of model recognition. Therefore, this study can use the clustering method to clear the data before training, eliminate useless interference data, and optimize the accuracy of model recognition.

In the construction of smart agriculture, the volume of data of edge equipment and the demand for data transmission are massive. Using edge computing to analyze edge data can effectively deal with data explosion and reduce network traffic pressure. In this study, a lightweight citrus flower recognition model was deployed using an intelligent station for citrus flower edge detection. This method reduces the data flow from the equipment to the cloud data center, shortens the response time of the equipment, realizes real-time identification, and provides a certain reference for the construction of intelligent agriculture.

### 4.2. Conclusions

This study proposes an improved YOLOv4 method to estimate the number of citrus flowers. We replaced MobileNetv3 with CSPDarknet53 in the backbone network and replaced deep separable convolution with standard convolution in the neck. The resultant model has a smaller footprint, lower overhead, and comparably higher accuracy.

(1) The improved YOLOv4 can maintain the detection performance of the original YOLOv4. In the case of sparse and medium citrus flowers, mAP can reach 95.5% and 88.1%, the number of weights is compressed by four times, and the detection speed is increased by 87%, indicating that the improved YOLOv4 can adapt to different scenarios and has high robustness.
(2) The deployment experiment shows that the speed of video stream detection on Nvidia Jetson AGX Xavier reached 11.3 FPS, indicating that the improved YOLOv4 has a smaller overhead. Compared with YOLOv4-tiny, the proposed method can also satisfy the practical requirement, while its mAP was 20.42% higher than the YOLOv4-tiny algorithm.

## Figures and Tables

**Table 1:** Improved YOLOv4 ablation experiment.

Network Model	Mean Average Precision/%	Parameter	Detection Speed/FPS	Weight/MB
YOLOv4	87.4	63,943,071	6.2	244
YOLOv4 + dw	85.8	35,690,655	11.1	136
YOLOv4 + dw + tiny	64.42	5,918,006	23.5	22.4
Improved YOLOv4 (YOLOv4 + dw + mobileNetv3)	84.84	11,309,039	11.6	53.7

**Table 2:** Comparison of detection results of different models.

Network Model	Mean Average Precision/%	F1 Score/%	Detection Speed/FPS	Weight/MB
YOLOv4	87.4	87.0	6.2	244
Improved YOLOv4	84.84	81.0	11.6	53.7
YOLOv4-tiny	64.42	61.0	23.5	22.4
Faster R-CNN	90.27	91.0	2.3	108.0

**Table 3:** Test results under different citrus flower densities.

Density	mAP@0.5/% Mean Average Precision	F1 Score/%
Few	87.4	87.0
Middle	84.84	81.0
Intensive	64.42	61.0
